# Stinging Stigma in Healthcare Symposium: A Participatory Approach to Disseminating Research and Strengthening Community

**DOI:** 10.35844/001c.161623

**Published:** 2026-06-08

**Authors:** J. Richelle Joe, Andrea Dunn, Shan-Estelle Brown, Makella Coudray, Cassandra Davis, Delrae Hartsfield, Whitney Marshall, Mikaela Mendoza-Cardenal, Dreysa Skinner, Ida Starks, Tiesha Williams, Karen Wint

**Affiliations:** 1 Department of Counselor Education and School Psychology, University of Central Florida,; 2 Let’s Beehive! Inc.,; 3 Rollins College,; 4 University of Central Florida,; 5 Stinging Stigma Planning Committee

**Keywords:** research dissemination, community-engaged, HIV

## Abstract

Typical research dissemination involves peer-reviewed publications and professional presentations targeting scholarly audiences. However, community-engaged health equity research requires innovative and community-centered dissemination approaches to ensure that research findings reach affected individuals and communities. Community-engaged research conferences are one such approach. This article provides guidance for planning and delivering a community-engaged research conference and uses the Stinging Stigma in Healthcare Symposium as an exemplar. This two-day event for healthcare providers and community members facilitated the dissemination of research focused on the sexual health and HIV prevention needs of Black women in Orange County, Florida. We discuss the formation of a planning committee, the steps in the planning process, and recommendations for researchers interested in using a symposium to disseminate their research.

Dissemination of community-engaged research to key stakeholders is necessary to ensure that scientific discoveries are used to develop policy and inform practice (Ashcraft et al., 2020; Gollust et al., 2025). Yet, gaps in translating research into practice exist, in part due to limitations in traditional methods of dissemination (Bodison et al., 2015; Gollust et al., 2025; Stewart et al., 2023). Research and dissemination are typically researcher driven, with the desired outcomes being published articles in peer-reviewed academic outlets (Bodison et al., 2015). However, jargon, paywalls, and densely written articles inhibit broad consumption of research, and even dissemination to non-academic audiences can be “unidirectional and not presented in an accessible format” (Bodison et al., 2015; Stewart et al., 2023, p. 92). Researchers bear the responsibility of developing proactive plans for ongoing dissemination that employ multiple strategies of engaging end users (Gollust et al., 2025; McBride et al., 2008). Among such strategies are research and policy briefs, townhalls, newsletters, mass media (e.g., radio, television), and social media, each designed to raise awareness, facilitate engagement or encourage advocacy and action (Gollust et al., 2025; McBride et al., 2008; Stewart et al., 2023).

Whereas academic conferences and symposia are typical methods by which experts share their research with other experts, community-engaged research conferences serve as solutions to the barriers of research uptake by community members (Bodison et al., 2015; Khodyakov et al., 2014). Such conferences require transparent, jargon-free communication from researchers to ensure accessibility. Additionally, they return research findings to the community so that the research is applicable and not merely extractive. Community-engaged research conferences require on-going engagement with community stakeholders, making them a natural outgrowth of community-based research projects. The purpose of this article is to provide a process for planning and delivering a community-facing research symposium designed to disseminate research findings. The *Stinging Stigma in Healthcare Symposium* will be used as an illustrative case of community-engaged research dissemination to address a critical health concern: HIV among Black women in Orange County, Florida.

## Stinging Stigma in Healthcare Symposium

**Figure 1.**
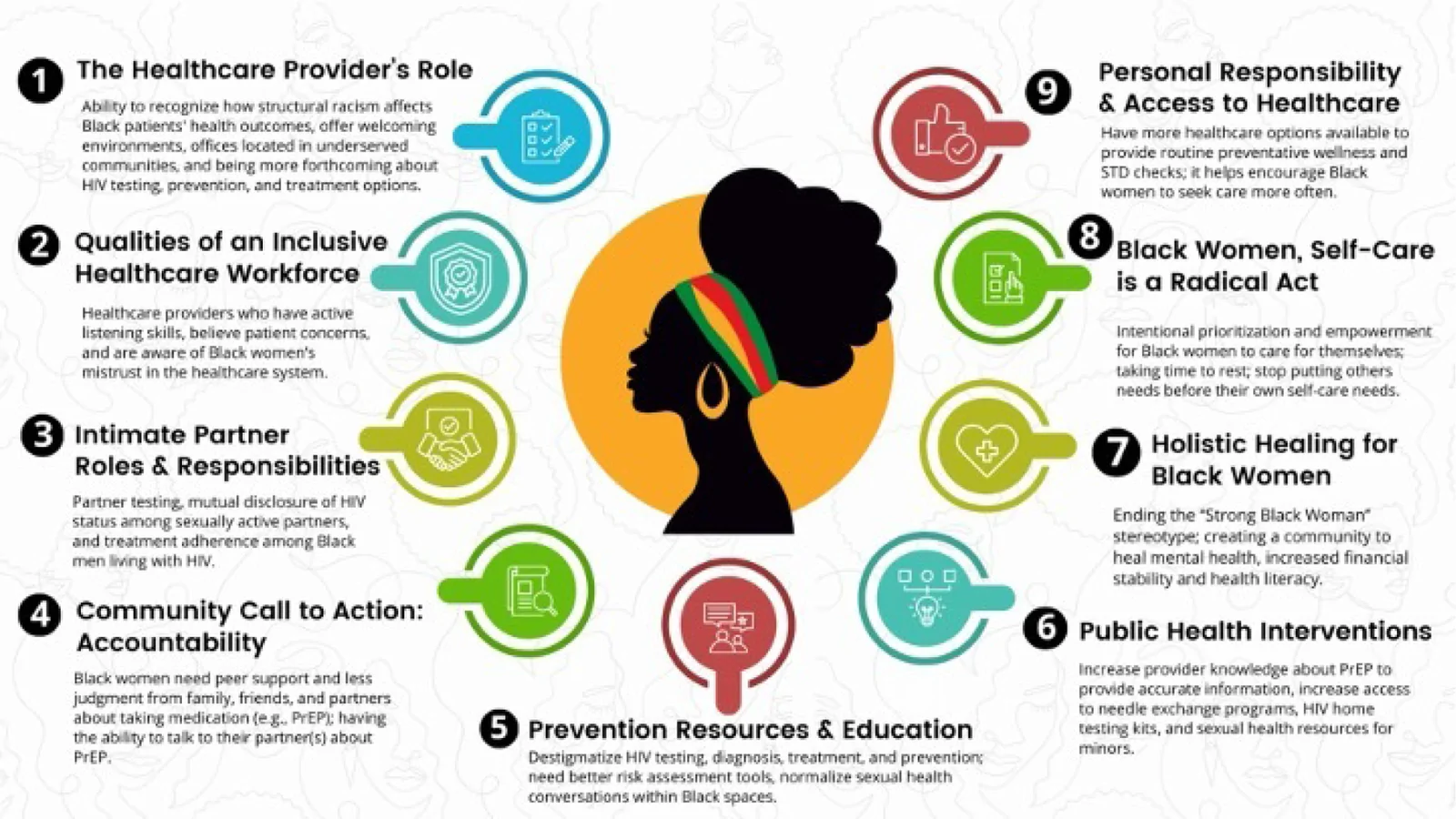
Nine Key Areas for Supporting Black Women’s Sexual Health

The National HIV/AIDS Strategy (The White House, 2021) and the Ending the HIV Epidemic (EHE; Department of Health and Human Services, 2026) initiative identify Black cisgender women and Orange County, Florida, as priorities for HIV prevention and intervention. Within Orange County, Black women are disproportionately affected by HIV, yet few HIV campaigns focus on them and their unique sexual health and HIV prevention needs. To address this service and research gap, a collaborative interdisciplinary team conducted a community-engaged research project to explore the factors associated with HIV vulnerability among Black women in Orange County, Florida (Joe et al., 2024, 2025). The study included group concept mapping, in-depth individual interviews with local women and providers, and consistent community stakeholder involvement in the research to yield findings with implications for healthcare providers, public health workers, and community members invested in Black women’s health. Data visualizations from the study indicated nine key areas critical to supporting Black women’s sexual health and HIV prevention efforts (see Figure 1; Joe et al., 2025).

To disseminate these findings, the research team, known as Team Orlando, collaborated with stakeholders to plan and deliver a public two-day symposium for healthcare providers and community members. Objectives for the symposium included developing a comprehensive plan for the symposium, effectively marketing the symposium, evaluating the program’s outcomes, and reviewing the evaluation data from the event. All objectives were met, and the *Stinging Stigma in Healthcare Symposium* took place February 7–8, 2025, with strong attendance from healthcare providers and community members. The two-day symposium was designed by the planning committee with the goal of moving away from the traditional academic conference model toward a more interactive, human-centered experience that reflected the lived realities and needs of Black women in Orange County, Florida. Every aspect of the symposium, from the agenda and environment to the choice of speakers and activities, was informed by the guiding principle of shared ownership between researchers, healthcare providers, and community members. The goal was not only to communicate research outcomes but also to foster ongoing dialogue, empathy, and collaborative learning around the stigma that drives HIV vulnerability among Black women.

## Planning a Community-engaged Symposium for Research Dissemination

Planning for a community-engaged research symposium requires continuous involvement and input from community stakeholders as well as specific logistical steps that ensure movement toward the desired goal. Important steps include committee formation, idea generation, and finalization of an event plan to be executed. Below, each step will be discussed along with real-world examples from the *Stinging Stigma in Healthcare Symposium*.

### Planning Committee Formation

Forming a planning committee is a critical first step that ensures meaningful involvement of both researchers and community members. Efforts should be made to include a diverse group of committee members who bring lived experience and expertise in multiple areas relevant to the research topic and the delivery of a large-scale event. Important considerations during this step include the time commitment and the possibility of compensation for the time committee members will devote to the planning process. Logistics such as day, time, and location should be considered with the assumption that prospective committee members will have competing obligations (e.g., work, childcare etc.). A schedule should be created and adhered to so that committee members are aware of the time commitment and researchers can be alerted of committee members’ availability. Additionally, care should be taken to establish rapport among committee members so that the group is able to work collaboratively to complete required tasks.

The formation of the planning committee for the *Stinging Stigma in Healthcare Symposium* overlapped with the conclusion of the research project as community members who participated in the study suggested the symposium and volunteered to assist with planning. The planning committee members were a diverse group with a wide range of knowledge, skills, and experiences relevant to the planning process. Members included employees at local HIV-serving organizations, individuals with event planning experiences, a professional baker who supplied custom baked goods for the event, and experts in health communication. Knowledge about the topic varied, but all committee members expressed a commitment to Black women’s sexual health. To establish collegiality within the group, community agreements that were used during data collection and analysis were revisited in the first planning meeting, and time was used to allow the group to reconnect and reformulate for the purpose of planning the symposium. Although the committee was focused on planning the event, there were moments of levity and wellness check-ins to address the heaviness of discussing Black women’s sexual health. Additionally, there were educational moments where committee members were able to learn throughout the process and share their insights with friends and family.

“Before joining the planning committee, conversations about knowing our status and sexual health were almost nonexistent in my circle. But after each session, I left feeling inspired—there was always something meaningful to take back and share with my friends. Being part of the process didn’t just inform me; it sparked deeper conversations and a stronger sense of ownership over our health within my community.”

- Planning Committee Member

Multiple elements of the planning meetings contributed to the effectiveness of the collaborative planning process. First, Team Orlando shared a structured meeting schedule with planning committee members prior to the first meeting and used electronic calendar invitations as reminders. Additionally, the day and time for the meetings were consistent over the five-month planning period, allowing the meetings to become part of committee members’ weekly routines. Clear objectives guided each meeting to encourage focus and productivity, and action steps were used to connect meetings and move the planning process forward. Examples of objectives include 1) identify skillsets among committee members, 2) brainstorm possible speakers, and 3) create event agenda. The use of incentives, both tangible (e.g., food, gift cards, and headshots) and intangible (e.g., interpersonal connection, learning opportunities, and resume-building experiences) deepened the committee members’ interest and engagement in the process.

### Idea Generation

The task of idea generation is a critical step once the planning committee has been formed. Foundational to this task is an understanding of the research topic and findings among the committee members. Here, researcher transparency undergirds the brainstorming process as aspects of the research and key findings are shared and discussed free of jargon. With an understanding of the research, the committee can then identify the primary goal for the event and supportive objectives that will lead to that goal. Brainstorming should include discussions of the event format, date, and location. Included with discussions of the event format should be potential speakers and methods of engagement with attendees. Additionally, brainstorming should include ideas regarding registration, incentives for attendees, branding, and marketing. Priorities for each element of the event should be community-driven, reflecting the expertise of individuals with lived experience in the target area.

**Figure 2.**
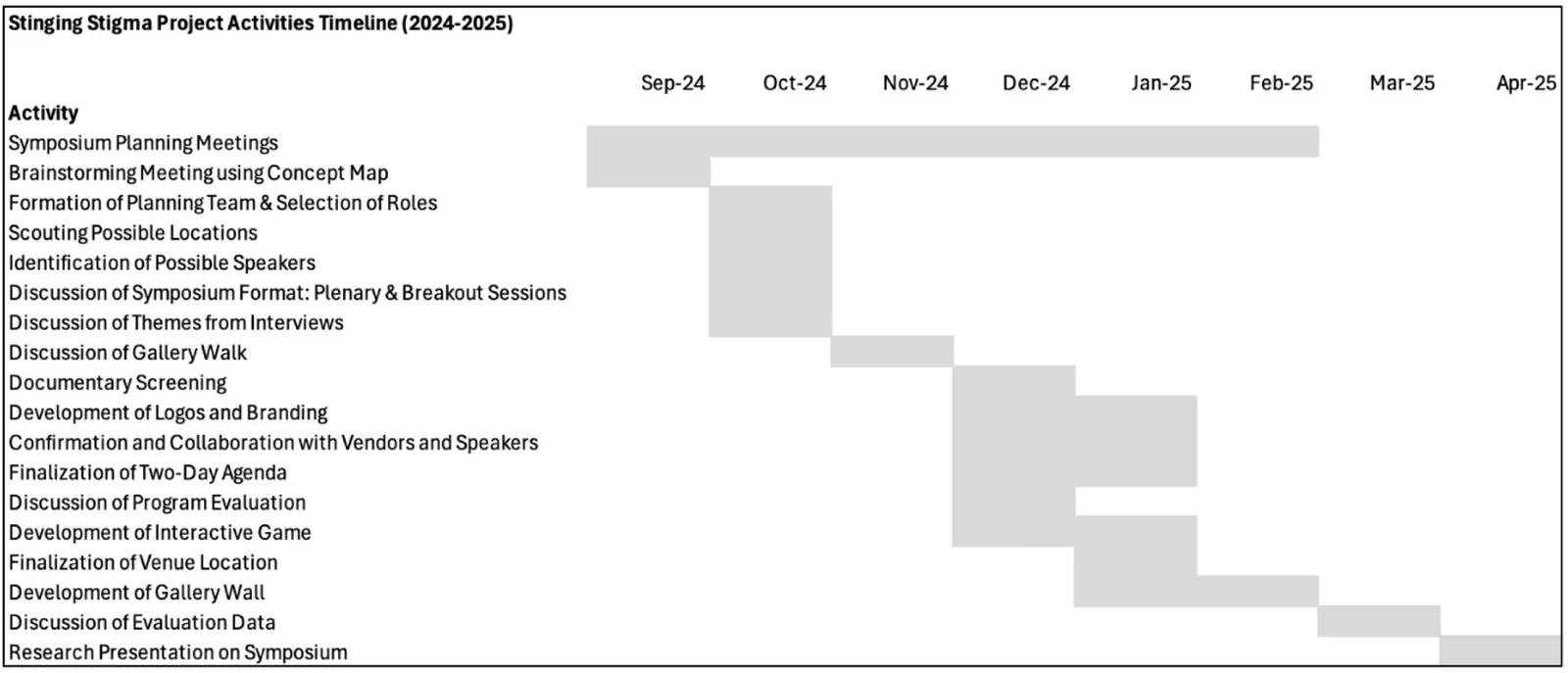
Stinging Stigma Project Activities Timeline (2024–2025)

To support brainstorming and establish common knowledge about what would be communicated at the *Stinging Stigma in Healthcare Symposium*, Team Orlando reviewed the research findings with the committee in the first planning meeting and used data visualizations throughout the planning process to remind committee members of the key findings to be shared. To incorporate additional information and context into the planning process, the committee members watched the documentary film *The Fight for Black Lives* (Keels, 2023) which addresses Black women’s health and health disparities. Once group dynamics were addressed and the planning committee was oriented to the study findings, brainstorming and the formulation of a clear plan for the symposium commenced. The planning committee met eight times over five months to plan the details of the symposium (see Figure 2).

The development of the event agenda and all activities occurred through collaborative dialogue and consensus-building. The committee began with an end goal in mind, discussing the desired aim, scope, atmosphere, and audience for the symposium. Strategic decisions were made regarding the length of the event in an effort to maximize attendance and minimize disruptions to potential attendees’ work and personal lives. The agenda went through multiple iterations as the committee discussed the event’s flow and imagined the experience for attendees. The committee generated a list of speakers and session facilitators, drawing from committee members’ personal and professional networks, and these speakers were aligned with aspects of the agenda that reflected their expertise. It is important to note that all ideas for the event were human-generated without the use of artificial intelligence to maintain closeness to the findings and community-engaged spirit of the research. Additionally, to ensure that the strategy for the symposium was feasible, an event planner was hired and invited to the committee meetings to provide guidance on the plan’s execution.

### Finalization of the Event Plan

The task of idea generation should result in an agreed-upon plan for the event. Consultation with a professional event planner is key throughout the planning process, but especially at this point given an event planner’s ability to determine feasibility of a plan. Details of the plan should include a confirmed date, time, and venue, which must be secured prior to invitations to speakers and vendors or marketing for the event. The size of the target audience, the format for the event, supply needs (e.g., technology and sound system), and the budget for the event all factor into venue selection and plan implementation. Expert advice from a professional event planner can assist with venue selection and anticipation of logistical needs for the event.

The planning committee decided that the *Stinging Stigma in Healthcare Symposium* would begin on February 7, 2025, a date chosen to acknowledge National Black HIV/AIDS Awareness Day (NBHAAD). The event venue was located in an accessible, well-known area of Orange County, and parking was compensated for the first 100 registrants. The planning committee paid careful attention to venue selection, ensuring sufficient size, accessibility, and professional support services. The space accommodated the large-group sessions and smaller breakout discussions reflected in the event agenda (see Tables 1 and 2). Technological readiness, including reliable sound equipment, screens, and projectors, supported seamless presentations. Venue staff and photographers maintained a high level of professionalism that contributed to the event’s smooth execution and credibility.

## Delivering a Community-engaged Symposium for Research Dissemination

During the delivery phase for a community-engaged research symposium, stakeholder engagement continues and even increases. As the event plan is implemented in partnership with community members, engagement with attendees, vendors, and other community partners should be intentional and reflective of the planning committee’s priorities. An evaluation at the conclusion of the event provides feedback for researchers, planning committee members, and speakers and can guide future dissemination efforts directed toward the community.

### Plan Implementation

The finalized plan for the event should be delivered in partnership with planning committee members, identified vendors and community partners, and other community members that have a vested interest in the research topic. The experience of attendees at the event should reflect the central focus of the research and the values of the planning committee. Although some elements of an academic conference may be retained (e.g., registration, speakers, breakout sessions), a community-engaged research symposium should look and feel different so that members of the community feel welcome and find the information accessible.

The *Stinging Stigma in Healthcare Symposium* was structured to prioritize audience engagement and experiential learning. The two-day format allowed attendees to build rapport, reflect deeply on complex issues, and return on the second day prepared for solution-oriented conversations. Day one was hosted by a local celebrity, radio DJ Monica May, and consisted of a gallery walk, dinner, and low-stakes interactive engagement with attendees (see Table 1). Event attendees began checking in at 6:00 pm and received a lanyard with a name badge and a red ribbon pin upon entrance. Attendees were then encouraged to visit vendor tables, network, and view the gallery wall of photos as dinner was served. Vendor tables included local HIV non-profit organizations, medical clinics, and educational institutions, all of which supported health and wellness in alignment with the research focus. Since the contracted event planner was responsible for venue logistics, planning committee members had the opportunity to network and ensure the environment was welcoming, comforting, and nonjudgmental.

The official program began at 7:00 pm with a formal welcome and an interactive game about HIV and PrEP basics that was facilitated by a planning committee member. Following the game, committee members were each assigned to a table to serve as research ambassadors, facilitating an activity based on the results of the initial research project. Quotes from Black women who were interviewed for the study were shared at each table, and planning committee members led discussions about the meaning of these quotes for community members and healthcare providers. Small group discussions were followed by a larger group discussion where insights from each table were shared with all in attendance. To close out the evening, attendees were led through a brief guided meditation activity and encouraged to return the following day prepared to engage deeper with the findings of the research study.

Day two of the symposium began with check-in and breakfast at 9:30 am, followed by plenary and breakout sessions throughout the day (see Table 2). One distinctive feature was the inclusion of two parallel tracks: one for healthcare providers and another for community members. This structure allowed each group to engage with material tailored to their specific contexts while also coming together in joint sessions that encouraged mutual understanding. Providers explored strategies for stigma reduction, trauma-informed care, and culturally responsive communication. Meanwhile, community participants discussed empowerment, advocacy, and navigating healthcare systems. Joint plenary sessions reconnected the groups to emphasize the shared responsibility and collective progress necessary for dismantling stigma. Sessions were designed to alternate between plenary discussions, breakout groups, and interactive panels. The planning committee served as facilitators throughout the event, ensuring that the audience remained active participants rather than passive recipients of information.

**Figure 3.**
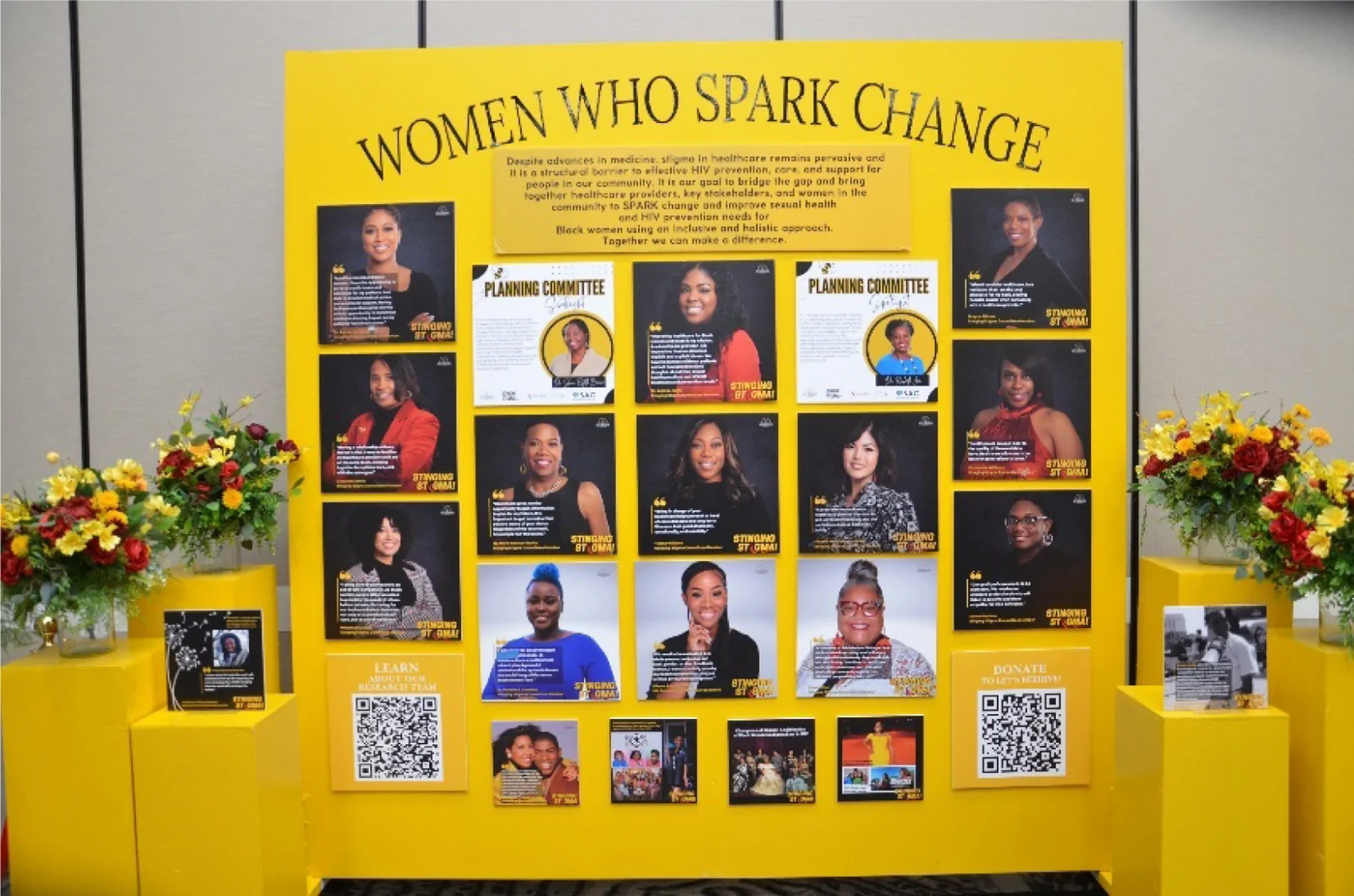
Stinging Stigma in Healthcare Gallery Wall

Visuals and branding also played a key role in shaping the atmosphere. The symposium’s use of vibrant imagery (see Figure 3) and affirming language centered on the strength and resilience of Black women. Pop-up banners relaying the nine key areas identified from the concept mapping research were placed throughout the hallways and main conference room. Signage and décor emphasized community, rest, and restoration rather than pathology or deficit. The attention to aesthetics and tone communicated that the symposium was a celebration of empowerment and partnership, not a clinical exercise.

### Audience Engagement and Priority Elements

The delivery of a community-engaged research symposium should prioritize bidirectional information sharing through immersive experiences that translate research findings into accessible and usable components. Speakers should be mindful not to simply talk at attendees but engage them in dialogue. Sessions should facilitate application of information to real-world experiences and encourage question-asking from attendees. Moreover, the atmosphere in the venue should reflect the topic of study, key insights, and values embodied by the planning committee and target community.

With recognition of the emotional labor and time required to engage in discussions about stigma and health inequities, the planning committee for the *Stinging Stigma in Healthcare Symposium* chose to intentionally incorporate wellness and appreciation elements throughout the symposium. Participation was free of charge, reflecting a commitment to accessibility and equity. Attendees received yoga mats and self-care gift boxes (rather than a conference bag or portfolio) that included informational materials, condoms, and other items to support wellness. Additionally, a select group of early registrants were designated “VIBees” to reflect the bee-themed branding of Let’s Beehive! Inc., the partnering non-profit organization on this project. These individuals received a complimentary copy of *Rest is Resistance: A Manifesto* by Tricia Hersey (2022). The selection of this book was intentional, as it underscored the importance of restful reflection and healing for both community members and professionals working in emotionally demanding fields. Desserts were provided by a small business owner who was also a member of the planning committee, offering an opportunity to support local entrepreneurship and reinforce community ownership of the event. These intentional gestures contributed to an environment where attendees felt valued and cared for.

The symposium’s emphasis on participatory engagement was most visible in its facilitation style and storytelling elements. A centerpiece of the event was the panel discussion during the lunch plenary on day two that featured women living with HIV. This panel humanized HIV and dismantled stereotypes by giving a face to the research findings. Women with lived experience shared their stories of navigating healthcare systems that often stigmatize or overlook Black women. Their narratives transformed abstract concepts such as intersectional stigma and healthcare inequity into tangible experiences that resonated with attendees. For providers, this represented an opportunity to confront implicit biases and reflect on how clinical environments can either perpetuate or disrupt stigma. For community members, it affirmed that their voices and experiences are vital to shaping effective, compassionate care.

*“As a heterosexual African American woman living and thriving with HIV, it is always an honor to be included in the planning and execution phases of events like the Stinging Stigma symposium, as inclusion not only highlights a frequently overlooked community but also provides an opportunity to showcase the individual behind the diagnosis*.”

– Planning Committee Member

Interactive elements extended beyond the panel. Each breakout session began with prompts that invited audience reflection on the interplay of personal biases, community experiences, and systemic barriers related to HIV care. Workshops incorporated role-playing exercises and small-group problem-solving to assess shifts in knowledge and attitudes. These activities created opportunities for honest dialogue across roles and disciplines. By encouraging participants to actively co-construct meaning, the design of the workshop sessions exemplified the project’s participatory ethos by translating the research findings into a collective learning experience.

### Vendor and Partner Engagement

Similar to vendor expos at academic conferences, a community-engaged research symposium can incorporate organizations, businesses, and other resources for the community that are relevant to the target audience. Incorporating vendors can offset some of the event’s costs and provide relevant information and services to attendees. For an event focused on health research, the engagement of vendors might mirror a community health fair, an established method of bridging healthcare providers and community members. Selection of vendors and partners might be open to all interested or it might be more selective to ensure consistency between the research being shared and the information and services provided by vendors. Determining which approach to take to vendors should be at the discretion of the planning committee so that the decision reflects community needs.

Vendor participation for the *Stinging Stigma in Healthcare Symposium* was curated with intention and alignment to the symposium’s mission. Rather than allowing open vendor registration, the planning committee extended invitations to organizations that shared a commitment to health equity and stigma reduction. Community-based organizations and public health partners staffed tables that provided attendees with culturally relevant resources. A notable feature was the inclusion of on-site HIV testing, which normalized testing as an act of empowerment and health maintenance, rather than a clandestine experience of shame and guilt. The diversity of participating groups illustrated the collaborative spirit at the heart of the symposium. Partners included local nonprofits, healthcare institutions, advocacy coalitions, and small businesses. Their presence reinforced the notion that addressing stigma and promoting health equity requires multisector collaboration, bridging clinical practice, public health, and community action.

### Event Evaluation

The evaluation of a community-engaged research symposium differs vastly from typical methods used to evaluate research dissemination. Rather than journal impact factor or citation count, evaluation data from a symposium relies on the perceptions of end users of the research. Information regarding their experience at the event, the value of the information shared, and its useability provide a rich picture that researchers and the planning committee can use to determine whether or not the event was delivered as intended. Both quantitative and qualitative evaluation data should be collected, and the instrument used to collect this data should be developed in partnership with the planning committee rather than solely developed by the researcher.

At the end of the closing session of the *Stinging Stigma in Healthcare Symposium*, attendees were asked to complete an electronic survey that asked both closed and open-ended questions about their experience at the event. Attendees were encouraged to complete the survey prior to leaving and were incentivized to do so with a custom-made baked good that matched the color scheme and theme of the event. Attendees’ evaluations illustrated the value of the event to healthcare providers and community members. The intentionality of the symposium’s delivery transformed research dissemination into a community-building event. The interactive structure and focus on shared experience made the findings tangible and actionable for attendees. Healthcare providers left with renewed insight into stigma’s complexity and were empowered with strategies for reducing their presence in clinical settings. Community members left feeling seen, respected, and equipped with tools for advocacy and self-care. The process of delivering the *Stinging Stigma in Healthcare Symposium* exemplified how participatory dissemination can strengthen both research impact and community solidarity by turning data into dialogue and dialogue into strategies for self-determination.

“As an academic researcher, participating in the planning committee has changed how I plan and conduct my research. I have updated my approach to community engagement, dissemination, and budget planning. The symposium set the standard for the quality of work I would like to produce moving forward.”

- Planning Committee Member

### Follow-up

Following the conclusion of a community-engaged research symposium, there should be re-engagement with the planning community and a debrief of the event to discuss impact and next steps. Event evaluations should be reviewed and discussed to ensure that the stated goals and objectives for the event were met. Insights from planning committee members about what they observed and experienced at the event are also valuable that this juncture and offer a different perspective from that of attendees. Additionally, debriefing and follow-up allow for discussions of sustainability, future research, and other ways to connect the community with research.

A debrief meeting was included in the planning meeting schedule for the *Stinging Stigma in Healthcare Symposium* and held within two weeks of the event. The purpose of the meeting was to review the evaluation data collected from symposium attendees and discuss committee members’ observations and experiences at the symposium. During the meeting, committee members’ shared anecdotes about their engagement with attendees as well as their learning experiences during panels and breakout sessions. This meeting also allowed for discussions about possible follow-up events and improvements that could inform future events. Additionally, the planning committee discussed what continued engagement might be for those who were interested in remaining connected and involved in HIV prevention and awareness work in Orange County, Florida. One member created a WhatsApp group chat to facilitate continued communication and sharing of information and local events relevant to the group. The group chat was integral to organizing the committee members to write this article about the *Stinging Stigma in Healthcare Symposium*. Both the group chat and the inclusion of committee members in the writing process for research dissemination are emblematic of the type of continuous engagement that is integral to community-engaged scholarship.

From the very start, I felt seen and heard. What began as planning an event for the community turned into finding a community within it.

- Planning Committee Member

## Recommendations for Researchers

Team Orlando disseminated their research findings via multiple outlets, such as peer-reviewed journal articles, professional presentations, and research briefs with the symposium being the method used to reach the audiences that provide services and support to Black women in pursuit of sexual healthcare. For researchers conducting community-engaged research or research that has particular relevance to community members and healthcare providers, a community-engaged research symposium like the *Stinging Stigma in Healthcare Symposium* might be an option to bridge the research-community dissemination gap (Bodison et al., 2015; Khodyakov et al., 2014). As such, the research team and the planning committee offer several recommendations for this type of dissemination (see Appendix), which are categorized by phase and corresponding intended outcome. It is important to note that the recommendations begin with the research itself and emphasize the importance of creative, participatory research designs and methods to ensure closeness to the communities and populations most impacted by the research. Such approaches to research reflect principles of human-centered design with the foregrounding of human needs and participation (Göttgens & Oertelt-Prigione, 2021). Moreover, community-based participatory research, such as that conducted by Team Orlando and disseminated by means of the symposium, acknowledges the expertise of multiple stakeholders, including those with lived experience, and allows for reciprocal learning and shared decision-making (Viswanathan et al., 2004). The broad desired aim of this type of research and dissemination is that relevant research findings reach affected individuals to advance health equity and social change.

## Conclusion

The *Stinging Stigma in Healthcare Symposium* brought together healthcare providers and community members to hear Black women’s voices and learn about the sexual health and HIV prevention needs of this often overlooked population. The symposium itself was a community-driven effort designed to return the research findings to the community rather than solely disseminate them through academic publishing. Engaging in this type of dissemination was a learning process for the research team, the committee members, and the attendees of the symposium. The experience transcended typical data collection and reporting of results to elevate the voices of the community and individuals with lived experience with HIV. Consequently, a key question following the symposium became “How can we keep this going?” This interest in following research with action reflects the commitment of the Robert Wood Johnson Foundation’s Interdisciplinary Research Leaders (n.d.) program to establish and maintain a Culture of Health. By conducting research in partnership with the community and disseminating the findings through the symposium, Team Orlando and the Stinging Stigma Planning Committee have contributed to a Culture of Health in Orange County, Florida and will continue this work to end the HIV epidemic among Black women in the region.

## Figures and Tables

**Table 1.** Day 1 Agenda

Time	Activity
6:00–7:00 p.m.	Registration Reception HIV testing (throughout) Gallery walk and vendor tables Welcome - overview of the research project and the purpose of the event
7:00–8:30 p.m.	HIV and PrEP Basics - Interactive game (Kahoot Game) Black Women’s Voices - quotes and discussion
8:30–8:45 p.m.	Closing - yoga/guided meditation/visioning/mindfulness
8:45–9:30 p.m.	Visit vendor tables

**Table 2.** Day 2 Agenda

Time	Activity		
9:30–10:00 a.m.	Attendees arrive and get breakfast		
10:00–10:45 a.m.	Opening Plenary- Holistic Healing for Black Women		
5 min Transition			
		Providers	Community
10:50–11:20 a.m.	Breakout 1	Engaging with healthcare	Galvanizing community
5 min Transition			
11:25–11:55 a.m.	Breakout 2	Public health efforts	Engaging with healthcare
5 min Transition			
12:00–1:00 p.m.	Lunch Panel- Black Women, Self-care is a Radical Act		
5 min Transition			
1:05–1:35 p.m.	Breakout 3	Galvanizing community	Public health efforts
5 min Transition			
1:40–2:30 p.m.	Closing Plenary - Personal Responsibility and Access to Healthcare		

## Appendix

**Table:**

Recommendations for Using a Symposium to Disseminate Research Findings		
Phase	Action	Intended Impact
**Data Collection**	Use innovative research methodologies (e.g., group concept mapping), including recruitment methods that cast a wide net and bring the research team closer to the community (e.g., attend a community festival)	Diverse perspectives Increased creativity Depth of understanding, Community buy-in No limits on who will have input Community members can find their strengths
**Planning**	Use meeting notes and recordings Budget for and hire an event planner Assemble a diverse planning committee Budget for committee member incentives (e.g., gift cards, food) Be intentional when selecting speakers, session facilitators, vendors, and sponsors Meetings with speakers prior to the event Create speaker agreements Recognize and leverage human connections and lived experience	Accuracy of the plan to be delivered Assistance with logistics and organization Various perspectives and strengths Engagement of the committee Ensures full alignment with the purpose of the research project All involved are aware of the research findings Accountability and clarity for all parties. Formalization of process Authenticity and human-centered design
**Delivery**	Lean on the event planner Delegate tasks to committee members and volunteers Audiovisual or IT person on site Intentional selection of a host with relevant knowledge and skills Develop an evaluation tool and collect evaluation data Make a call to action	Ensures expertise in delivery, attendee engagement, set-up, breakdown and flow Ensures flow and engagement of the committee based on strengths Manage tech issues Engagement and professionalism Determine if goals/outcome objectives were met and identify improvements Build on momentum to continue advocacy beyond the event
**Post Symposium Engagement**	Review evaluation data and conduct a SWOT analysis Follow up with attendees Highlight photos/video	Inform next steps and decision-making regarding a follow-up event Maintain connection and momentum
